# Effects of wheat germ diet on intestinal antioxidant capacity, immunological function and gut microbiota of Sichuan white geese

**DOI:** 10.3389/fmicb.2024.1435454

**Published:** 2024-09-11

**Authors:** Xin Wang, Dongmei Jiang, Xiaoguang An, Shuo Li, Yuxin Qi, Yujie Yang, Zelong Wang, Qian Sun, Weikang Ling, Chengweng Ji, Yuxuan Qi, Hengyong Xu, Chunchun Han, Hua Zhao, Bo Kang

**Affiliations:** ^1^State Key Laboratory of Swine and Poultry Breeding Industry, Farm Animal Genetic Resource Exploration and Innovation Key Laboratory of Sichuan Province, College of Animal Science and Technology, Sichuan Agricultural University, Chengdu, China; ^2^Animal Nutrition Institute, Sichuan Agricultural University, Chengdu, China

**Keywords:** Sichuan white geese, wheat germ diet, intestinal flora, intestinal barrier, intestinal health

## Abstract

**Background:**

Wheat germ is known for its antioxidant, anti-inflammatory, and disease resistance properties in animals. However, its effect on the gut of Sichuan white geese remains unclear.

**Method:**

In this study, thirty 250-day-old geese were divided into three equal groups, the control group, LWG group (21.8% wheat germ) and HWG group (43.6% wheat germ), the experiment lasted 12 weeks. We assessed various aspects of geese intestinal health, including barrier function, digestibility, antioxidant capacity, immunity, microbiota, and metabolism.

**Results:**

The study revealed a significant increase in villus height (VH), villus height-to-crypt depth (VH/CD) ratio, amylase, and lipase activities in the duodenum and ileum, increased putrescine levels in the duodenum and jejunum, as well as spermidine levels in the jejunum (*P* < 0.05). LWG increased the total antioxidant capacity (T-AOC) in the duodenum, while decreasing levels of intestinal malondialdehyde (MDA), serum lipopolysaccharide (LPS), interleukin-6 (IL-6), and diamine oxidase (DAO) activity (*P* < 0.05). Furthermore, LWG increased the relative abundance of *Oscillospiraceae_unclassified*, *Ligilactobacillus*, and *Roseburia*, as well as increased levels of acetic acid, butyric acid, and valeric acid, while decreasing the relative abundance of *Subdoligranulum*, *Flavonifractor*, and *Klebsiella*. Additionally, we observed 17 up-regulated genes and 25 down-regulated genes in the jejunum, which are associated with the cell cycle and immunity. These genes play roles in pathways such as the p53 signaling pathway, cell cycle regulation, and pathways associated with immune modulation. On the other hand, HWG increased intestinal VH and spermidine levels, as well as amylase and lipase activities in the duodenum (*P* < 0.05). It also elevated ileal T-AOC and sIgA levels (*P* < 0.05), while reducing intestinal MDA content, serum LPS levels, DAO activity, and propionic acid in cecum contents (*P* < 0.05). Moreover, HWG increased the relative abundance of *Ligilactobacillus*, *Oscillospiraceae_unclassified*, and *Roseburia* (*P* < 0.05).

**Conclusion:**

Overall, wheat germ diets, particularly the LWG diet demonstrated the ability to enhance antioxidant capacity, digestibility, immunity, and barrier properties of the intestinal tract, while modulating the gut microbiota and metabolism. Therefore, wheat germ diets hold promise in improving intestinal health by preserving barrier function and regulating flora structure.

## 1 Introduction

The intestine plays a pivotal role as the host’s largest immune organ, significantly impacting the production capacity and overall health of animals (de Vos et al., 2022; Shan et al., 2024). Maintaining intestinal health involves several key factors, including complete gut morphology, a robust immune system, effective barrier function, and a stable microbial composition (Shehata et al., 2022). In addition to its primary function of digesting and absorbing nutrients in poultry, the gut serves as a natural defense mechanism, upholding the stability of the internal environment (Wang et al., 2013; Wen et al., 2024). A well-functioning intestinal barrier allows for the absorption of beneficial nutrients while effectively preventing the ingestion of harmful substances. Conversely, any compromise in the integrity of the barrier can result in the ingestion of pathogens and toxins, disrupting the delicate balance within the intestine (Khoshbin and Camilleri, 2020; Zhu La et al., 2024). Intestinal flora not only serves as a source of nutrients for the host and helps maintain intestinal homeostasis but also plays a crucial role in enhancing mucosal immune function, promoting the development and maturation of immune organs, and improving both specific and non-specific immune responses in animals (Jandhyala et al., 2015; Luthold et al., 2017).

Wheat germ, a by-product of wheat, constitutes approximately 2% to 3% of the total weight of wheat and boasts a wealth of bioactive components, including polyamines, carotenoids, tocopherols, flavonoids, policosanols, phytosterols, and other valuable compounds, which impart antioxidant and anti-inflammatory properties (Telekes et al., 2007; Naureen et al., 2022). In animal models, wheat germ has demonstrated promising anti-tumor properties and the ability to confer resistance against inflammation (Ojo et al., 2017). Notably, fermented wheat germ has shown potential in improving neurotransmitters in the hippocampus of depressed rats through the brain-gut axis. This fermentation process also remodels the structure of the intestinal flora, restores amino acid metabolism function, and ameliorates stress-induced depressive behavior in rats (Hu et al., 2023). Research has reported that daily intake of 300 mg/kg of wheat germ in the diet can significantly enhance the antioxidant capacity in the bodies of mice on a high-fat diet, and wheat germ has exhibited remarkable efficacy in reducing senile osteoporosis in elderly rats through the regulation of the OPG/RANKL/RANK/TRAF6 pathway and mitigation of oxidative stress levels (Wang et al., 2022; Liu et al., 2022). Additionally, a diet containing 10% wheat germ can reduce pro-inflammatory factors, increase the expression of intestinal tight junction proteins, improve intestinal barrier integrity, and alleviate intestinal inflammation through the IL-22 signaling transduction pathway and the pSTAT3 signaling pathway in mice with intestinal inflammation (Alake et al., 2023). In human studies, wheat germ has been found to significantly increase the activity of serum alanine aminotransferase and γ-glutamyltransferase, enzymes associated with hepatic steatosis, while improving total antioxidant capacity and reducing serum total cholesterol and triglyceride levels, thereby aiding in the attenuation of fatty liver degeneration (Salehi-Sahlabadi et al., 2022). In addition, wheat germ has demonstrated preventive effects against the development of colon cancer, breast cancer, and cardiovascular disease (Naureen et al., 2022). However, the effects of wheat germ on the intestinal barrier and flora structure of geese remain unclear. Therefore, this study intended to investigate the effects of wheat germ diets on the intestinal health of Sichuan white geese, with the objective of shedding light on potential strategies to enhance animals’ production performance and explore alternatives to antibiotic usage.

## 2 Materials and methods

### 2.1 Animals and diets, and experimental design

The experimental protocols involving Sichuan white geese were conducted in accordance with the guidelines provided by the Animal Ethics Committee of the College of Animal Science and Technology, Sichuan Agricultural University (permit number: DKY-B2021302175).

Thirty female Sichuan white geese, aged 250 days, possessing similar health status and weight, were randomly assigned to three groups, with ten geese in each group (*n* = 10). All geese were reared under identical environmental conditions in Ya’an, Sichuan, China, and had free access to drinking water. During the experiment, all geese were raised in a closed cage with a density of 0.5m^2^ per goose, they were fed three times a day, at 8:00 am, 14:00 pm, and 19:00 pm. The experiment ran for 12 weeks after 1 week of pre-feeding. The Control (Con) group was fed a corn-soybean meal basal diet (DM basis), while the Low Wheat Germ (LWG) group received a diet containing 21.8% wheat germ (Leader S249 Silver Star Powder Co., Led., Dezhou, China). The High Wheat Germ (HWG) group was provided with a diet comprising 43.6% wheat germ. The formulation of the feed, including the elemental diet composition and nutritional content, was designed based on the Chinese Feed Composition and Nutritional Value Table, as outlined in Table 1. The nutritional composition of the feed was kept consistent for all three groups of Sichuan white geese. The body weight was measured on day 0 and 84, and then the determination of average daily gain (ADG) was calculated. Total feed intake was recorded and then the average daily feed intake (ADFI) was calculated.

**TABLE 1:** Composition and nutrient levels of Sichuan white geese (DM basis).

Project	Con	LWG	HWG
**Ingredients(100%)**			
Wheat germ	–	21.80	43.60
Corn	41.84	41.41	42.20
Unite bran	11.00	6.40	2.32
Wheat bran	5.00	4.00	1.00
Soybean meal	20.00	10.00	1.00
Rapeseed meal	6.90	4.20	1.00
Soybean oil	6.30	3.20	–
Salt	0.30	0.30	0.30
Calcium hydrogen phosphate	1.40	1.15	1.05
Calcium carbonate	6.70	7.00	7.00
Methionine	0.06	0.04	0.03
Choline chloride	0.10	0.10	0.10
Premix	0.40	0.40	0.40
**Total**	100	100	100
**Nutrient level**			
Metabolizable energy	10.63	10.61	10.62
Crude protein	16.58	16.54	16.56
Calcium	2.89	2.89	2.81
Available phosphorus	0.37	0.36	0.37
Lysine	0.74	0.74	0.75
Methionine	0.32	0.31	0.31
Cystine	0.30	0.30	0.30
Threonine	0.63	0.63	0.63
Crude fiber	6.27	6.29	6.29

Premix provides the following per kilogram of diet: vitamin A 6000 IU, vitamin B_1_ 1.2 mg, vitamin B_2_ 2.4 mg, nicotinic acid 20 mg, vitamin B_6_ 3 mg, vitamin D_3_ 1500 IU, vitamin E 10 IU, vitamin K_3_ 2.4 mg, vitamin B_12_ 0.01 mg, biotin 0.15 mg, calcium pantothenate 10 mg, Fe 85 mg, Zn 80 mg, Cu 8 mg, I 1 mg, Mn 85 mg, Se 4.5mg.

### 2.2 Sample collection

At the end of the 12-week experimental period, for each group, blood samples were obtained from four randomly selected geese using the inferior pterygoid vein. The collected blood samples were then centrifuged at 2,000 × g for 15 min, and the resulting serum was stored at −80°C until further analysis. Subsequently, the geese were humanely euthanized by bleeding through the neck. The abdominal cavity was promptly opened, and the duodenum, ileum, and jejunum from three geese were fixed in 4% paraformaldehyde for subsequent histological examination. The remaining intestinal tissue from the remaining geese was carefully collected and stored at −80°C for further analysis.

### 2.3 Histomorphometry of intestinal tissue

To analyze the histomorphology of the intestinal tissue, the tissue specimens were initially washed with phosphate buffered saline to remove any residual blood and gently dried with filter paper. Subsequently, the fixed intestinal tissue underwent a dehydration process using a series of ethanol solutions with gradually increasing concentrations (75, 85, 90, 95, and 100%). The tissue samples were then equilibrated in xylene before being embedded. Following the dewaxing step, tissue sections with a thickness of 4–5 μm were prepared. To visualize the morphological features of the intestinal tissue, these sections were stained with hematoxylin and eosin (H&E). The stained tissue sections were observed under light microscopy equipped with a digital camera, and images were captured for further analysis.

### 2.4 Determination of polyamines

For the analysis of polyamines, 0.1 *g* of the weighed intestinal sample was homogenized using a glass homogenizer in 1.5 mL of 5% HClO_4_. The homogenate was then incubated at 40°C for 60 min to facilitate the benzoyl chloride derivatization of polyamines, following the method described by Kang et al. (2017). Subsequently, benzoylated polyamines were separated using a C18 extraction column. The analysis procedure was as follows: a mobile phase consisting of 62% methanol and 38% ultrapure water was used. Each sample was analyzed for a duration of 20 min, maintaining a constant temperature of 39°C for the extraction column. The flow rate was set at 1 mL/min, and the UV wavelength for detection was set at 229 nm. To ensure accuracy, the results were normalized using 1,6-hexanediamine (Sigma, USA) as the internal standard. Standard curves for putrescine, spermidine, and spermine (all from Sigma, USA) were utilized for quantification purposes.

### 2.5 Determination of short-chain fatty acids

An appropriate amount of cecal contents was weighted and thoroughly mixed with ultrapure water. The mixture was then allowed to stand still and subsequently centrifuged at 4°C for 10 min at 12,000 × g. The resulting supernatant was collected, and to this, 0.2 mL metaphosphoric acid and 23.3 μL of a 210 mmol/L crotonic acid solution were added. The mixture was left to stand for 30 min before being centrifuged again for 10 min at 8,000 × g. A 0.3 mL portion of the supernatant was collected and mixed with 0.9 mL of chromatographic methanol. The sample mixture was then subjected to filtration using a 0.22 μm membrane before being analyzed for short-chain fatty acids using a gas chromatograph.

### 2.6 Antioxidant capacity detection kit

The appropriate amount of intestinal tissue samples were weighed, and 1mL of ice-cold phosphate buffered saline was added for homogenization. The homogenate was then centrifuged at 12,000 × g for 5 min at 4 °C, and the resulting supernatant was collected and filtered through a 0.22 μm pore size filter. The protein concentration of the intestinal tissue homogenate was determined using the BCA detection kit (Beyotime, China), and the obtained protein solution was used directly for malondialdehyde (MDA) detection.

The Thiobarbituric Acid storage solution and working solution were prepared accordingly. The samples were added to the detection wells and heated at 100°C for 15 min. After cooling to room temperature, the samples were centrifuged at 1000 × g for 10 min, and 200 μL of the supernatant was transferred to a 96-well plate for detection.

The 2,2′-Azino-bis (3-ethylbenzothiazoline-6-sulfonic acid) master mix was prepared following the Total Antioxidant Capacity (T-AOC) instructions. The mixture was stored at room temperature, protected from light, for 14 hours. Trolox, a known antioxidant, was diluted to the appropriate concentration in phosphate buffered saline. The reagents were added to the corresponding wells of the plate, and the absorbance at 734 nm was measured. The MDA and T-AOC contents were then calculated based on the standard curve and protein concentration obtained.

### 2.7 Detection of blood indicators using ELISA kit

Prior to analysis, the serum samples were thawed at 4°C and thoroughly mixed. Enzyme-linked immunosorbent assay (ELISA) kits from Meimian Industrial Co., Ltd. (Jiangsu, China) were utilized to assess levels of Immunoglobulin A (IgA), Immunoglobulin M (IgG), IL-1β, IL-6, IL-10, amylase, lipase, trypsin, Lipopolysaccharide (LPS), diamine oxidase (DAO) in the serum, as well as intestinal secretory immunoglobulin A (sIgA). The ELISA kit instructions were followed accordingly. Specimens, standards, and HRP-labeled detection antibodies were added to pre-coated antibody microplates, followed by incubation, and washing, and color development using the substrate TMB. Subsequently, the absorbance was measured at a wavelength of 450 nm.

### 2.8 16S rRNA amplicon sequencing and bioinformatics

Intestinal contents of six geese in each group were randomly selected for 16S rRNA amplicon sequencing, DNA extraction from different samples followed the CTAB protocol as per the manufacturer’s instructions. The resulting total DNA was eluted in 50 μL of Elution buffer and stored at −80°C. The quality and integrity of each DNA sample were assessed by electrophoresis in a 1% agarose gel with Tris-acetate-EDTA (TBE) buffer. The DNA concentration was quantified using a spectrophotometer. PCR amplification was performed in a 25 μL reaction mixture containing 25 ng of template DNA, 12.5 μL of PCR Premix, 2.5 μL of each primer, and PCR-grade water to adjust the volume. The PCR products were confirmed by 2% agarose gel electrophoresis. The amplicon pools were prepared for sequencing, and the size and quantity of the amplicon library were assessed using the Agilent 2100 Bioanalyzer (Agilent, USA) and the Library Quantification Kit for Illumina (Kapa Biosciences, Woburn, MA, USA), respectively. The libraries were sequenced on the NovaSeq PE250 platform. The removal of chimeric and redundant sequences was performed in this step, followed by the selection of operational taxonomic units (OTUs) based on a 97% similarity threshold specific to the 16S rRNA V3-V4 region. Next, the OTUs were taxonomically annotated using the Silva (release 138) database. Sequence data analyses were mostly processed using QIIME2 and R package (v3.5.2). Alpha diversity (α-diversity) indexes, including Chao1 richness estimator, Shannon diversity index, Simpson index, observed species, and Goods coverage, were calculated in QIIME2 and displayed as box plots to reflect the richness and uniformity of cecal microbial communities. The structural variation of microbial communities among samples was investigated by beta diversity (β-diversity) analysis, which was carried out using UniFrac distance metrics, and the results were illustrated via principal coordinate analysis.

### 2.9 Transcriptome sequencing

RNA sequencing was performed on jejunal tissue from four randomly selected geese in each group. Total RNA was extracted and purified using Trizol reagent (Invitrogen, USA) following the manufacturer’s procedure. The quantity and purity of each RNA sample were determined using a NanoDrop ND-1000 spectrophotometer (NanoDrop, USA). RNA integrity was assessed using the Bioanalyzer 2100 system (Agilent, USA), ensuring a RIN number > 7.0, and confirmed by denaturing agarose gel electrophoresis. Paired-end sequencing (PE150) with a read length of 2 × 150bp was performed on an Illumina Novaseq™ 6000 platform (LC-Bio Technology CO., Ltd., China). To preprocess the sequencing data, the fastp software was employed to remove reads containing adaptor contamination, low-quality bases, and undetermined bases, using the default parameters. The reads were then mapped to the reference genome of the Sichuan white goose (GCA_002166845.1) using HISAT2. After the final transcriptome was generated, StringTie was used to estimate the expression levels of all transcripts, and FPKM was calculated as a measure of mRNA expression level. Differentially expressed mRNAs were identified based on a fold change > 1.5 or fold change < 0.5, and statistical significance was determined using a parametric F-test comparing nested linear models (*p*-value < 0.05) with the R package edgeR. Finally, both Gene Ontology (GO) and Kyoto Encyclopedia of Genes and Genomes (KEGG) functional enrichment analysis were performed using DAVID software.^1^

### 2.10 Statistical analysis

The statistical analysis of the data was performed using the general linear model program SPSS (SPSS Inc., Armonk, NY, USA). Data were tested for normality (Shapiro–Wilk test) and homogeneity of variance (Levene’s test) at a significance level of 5% prior to further analysis. Duncan multiple comparisons in one-way analysis of variance (ANOVA) were used to test the significance of differences between groups. with a significance level set at *P* < 0.05. The data are presented as means ± SEM, and each assay included a minimum of three independent samples. Graphs were generated using Origin software (Version 2023b, Northampton, MA, USA).

## 3 Results

### 3.1 Effect of wheat germ diet on body weight and feed intake

Table 2 presents the ADG and ADFI of the geese. There were no significant differences in ADG and ADFI among the treatment groups (*P* > 0.05).

**TABLE 2:** Effect of spermidine-rich diet on average daily gain and average daily feed intake.

Parameters	Con	LWG	HWG	SEM	P-value
Initial body weight/kg	3.87	3.66	3.78	0.10	0.623
Final body weight/kg	4.43	4.30	4.40	0.11	0.50
Average daily gain/g	6.90	7.62	7.38	0.48	0.16
Average daily feed intake	187.72	188.14	171.43	9.65	0.403

Effect of spermidine-rich diet on average daily gain and average daily feed intake Data are presented as the mean ± SEM (*n* = 10 per group). Different letters denote significant differences between groups (*P* < 0.05), while the same letters indicate no significant difference (*P* > 0.05).

### 3.2 Promoting intestinal development

**FIGURE 1:**
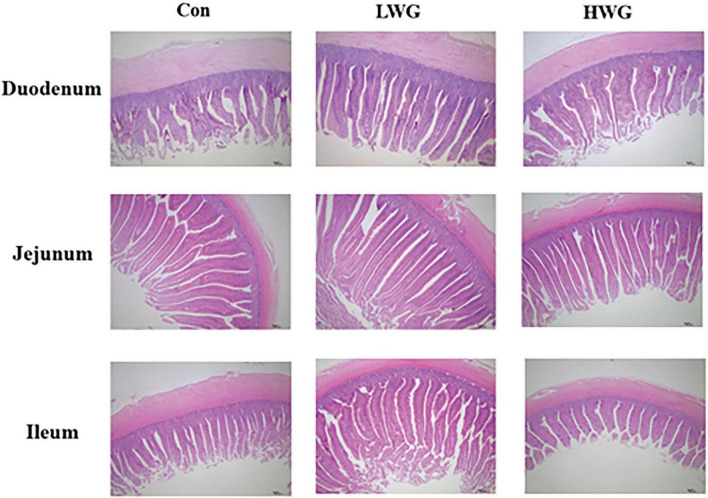
Effect of wheat germ diets on duodenal, jejunal and ileal morphology.

The effects of wheat germ on intestinal morphology changes are shown in Figure 1 and Table 3. Compared to the control group, the LWG group exhibited a remarkable enhancement in intestinal VH and VH/CD (*P* < 0.05). Additionally, the duodenum and ileum CD were decreased by 8.27 and 15.08%, respectively, while the jejunum CD remained unchanged (*P* > 0.05). In the HWG group, the intestinal VH was significantly increased by 10.12%, and CD in the duodenum and ileum was reduced by 14.40 and 12.44% (*P* < 0.05), respectively. Furthermore, the ileum VH/CD was significantly increased (*P* < 0.05), while the duodenum VH/CD remained unchanged (*P* > 0.05; Table 2).

**TABLE 3:** Effects of wheat germ on intestinal VH, CD, and VH/CD.

Para-meters	Con	LWG	HWG	SEM	*P*-value
**Duodenum**					
VH, μm	968.95^c^	1312.74^a^	1067.04^b^	28.18	< 0.001
CD, μm	281.00^a^	257.75^b^	240.53^b^	4.47	0.010
VH/CD	3.34^b^	4.13^a^	3.67^b^	0.10	0.010
**Jejunum** VH, μm	1581.68^b^	1911.37^a^	1884.79^a^	35.50	< 0.001
VH, μm	1581.68^b^	1911.37^a^	1884.79^a^	35.50	< 0.001
CD, μm	182.68	188.55	206.3	4.56	0.161
VH/CD	8.79^b^	10.38^a^	8.97^b^	0.25	0.015
**Ileum** VH, μm	1601.89^b^	1981.99^a^	2044.82^a^	50.00	< 0.001
VH, μm	1601.89^b^	1981.99^a^	2044.82^a^	50.00	< 0.0001
CD, μm	240.33^a^	203.06^b^	210.44^b^	5.26	0.009
VH/CD	6.11^b^	7.60^a^	7.42^a^	0.20	0.002

VH, Villus height, CD: Crypt depth, VH/CD: Villus height to crypt depth ratio. Data are presented as the mean ± SEM (*n* = 3 per group). Different letters denote significant differences between groups (*P* < 0.05), while the same letters indicate no significant difference (*P* > 0.05).

### 3.3 Increasing intestinal polyamine levels

The effect of wheat germ on intestinal polyamine levels is shown in Table 4. In comparison to the control group, the duodenal putrescine levels was significantly higher in the LWG group by 30% (*P* < 0.05), while spermidine and spermine levels remained unchanged (*P* > 0.05). In contrast, the levels of spermidine and spermine increased by 19.46 and 19.95% in the HWG group, and levels of putrescine and spermidine were elevated by 59.69 and 43.12% in the LWG group (*P* < 0.05), while spermine levels remained unaltered (*P* > 0.05). On the other hand, the levels of putrescine and spermine decreased by 23.58 and 12.67% in the HWG group (*P* < 0.05), along with an increase in spermidine levels (*P* > 0.05). In the ileum, the spermine levels reduced by 17.4% in the LWG group (*P* < 0.05), while putrescine and spermidine levels remained unchanged (*P* > 0.05). Conversely, the HWG group exhibited a significant increase in spermidine levels and a decrease in spermine levels (*P* < 0.05).

**TABLE 4:** Intestinal putrescine, spermidine, and spermine contents.

Para-meters	Con	LWG	HWG	SEM	*P*-value
**Duodenum**					
Putrescine, mg/kg	28.43^b^	36.96^a^	28.59^b^	1.35	0.071
Spermidine, mg/kg	400.00^b^	454.01^ab^	477.83^a^	12.70	0.034
Spermine, mg/kg	448.28^b^	652.82^ab^	559.99^a^	25.72	0.090
**Jejunum**					
Putrescine, mg/kg	32.57^b^	52.01^a^	24.89^c^	0.41	< 0.001
Spermidine, mg/kg	310.78^b^	444.79^a^	403.00^a^	37.88	< 0.001
Spermine, mg/kg	561.90^a^	527.72^a^	490.72^b^	49.14	< 0.001
**Ileum**					
Putrescine, mg/kg	17.55	20.58	17.59	0.74	0.335
Spermidine, mg/kg	205.85^b^	182.11^b^	285.51^a^	14.85	0.002
Spermine, mg/kg	334.64^a^	297.97^b^	276.42^b^	25.66	0.004

Effect of wheat germ diets on putrescine, spermidine, and spermine contents in the duodenum, ileum, and jejunum. Data are presented as the mean ± SEM (*n* = 3 per group). Different letters denote significant differences between groups (*P* < 0.05), while the same letters indicate no significant difference (*P* > 0.05).

### 3.4 Increasing intestinal digestive enzyme activity

The impact of wheat germ on intestinal digestive enzyme activity is shown in Table 5. Compared to the control group, wheat germ supplementation significantly increased the activities of amylase and lipase in both the duodenum and ileum (*P* < 0.05). In the LWG group, jejunal trypsin and lipase activities were significantly increased by 26.34 and 18.54% (*P* < 0.05), while the trypsin activity of the duodenum and ileum remained unchanged (*P* > 0.05). The HWG group showed no significant changes in trypsin and lipase activities in the jejunum (*P* > 0.05).

**TABLE 5:** Activity of intestinal Amylase, Trypsin, and Lipase.

Para-meter	Con	LWG	HWG	SEM	*P*-value
**Duodenum**					
Amylase, IU/L	13.30^b^	15.03^a^	14.89^a^	0.38	< 0.001
Trypsin, IU/L	7.19	7.18	7.20	0.15	0.870
Lipase, IU/L	31.42^b^	39.16^a^	42.17^a^	1.73	< 0.001
**Jejunum**					
Amylase, IU/L	19.65	19.79	19.88	0.30	0.025
Trypsin, IU/L	12.53^b^	15.83^a^	14.17^ab^	0.42	< 0.001
Lipase, IU/L	49.47^b^	58.64^a^	54.73^ab^	1.66	0.003
**Ileum**					
Amylase, IU/L	19.88^c^	23.00^b^	29.31^a^	1.34	< 0.001
Trypsin, IU/L	19.26	19.57	19.94	0.40	0.039
Lipase, IU/L	37.39^b^	48.73^a^	57.19^a^	2.66	0.007

Effect of wheat germ diets on lipase, amylase, and trypsin activities in the duodenum, ileum, and jejunum. Data are presented as the mean ± SEM (*n* = 4 per group). Different letters denote significant differences between groups (*P* < 0.05), while the same letters indicate no significant difference (*P* > 0.05).

### 3.5 Enhancing intestinal antioxidant capacity

The levels of oxidation and antioxidant markers in intestinal tissues were explored (Table 6). Compared to the control group, the LWG and HWG groups showed a remarkable decrease in duodenum, jejunum, and ileum MDA levels (*P* < 0.05). Regarding T-AOC, the duodenum T-AOC level was enhanced in the LWG group, while the ileum T-AOC level was increased in the HWG group (*P* < 0.05). However, T-AOC levels in the ileum and jejunum remained unchanged in the LWG group (*P* > 0.05).

**TABLE 6:** The leaves of MDA and T-AOC in the duodenum, ileum, and jejunum.

Para-meters	Con	LWG	HWG	SEM	*P*-value
**Duodenum**					
MDA, μmol/mg	0.13^a^	0.07^c^	0.10^b^	0.01	0.001
T-AOC, mmol/mg	0.48^b^	0.93^a^	0.55^b^	0.05	< 0.001
**Jejunum**					
MDA, μmol/mg	0.20^a^	0.15^b^	0.14^b^	0.03	< 0.001
T-AOC, mmol/mg	0.34	0.37	0.34	0.01	0.431
**Ileum**					
MDA, μmol/mg	0.38^a^	0.27^b^	0.16^c^	0.02	0.007
T-AOC, mmol/mg	0.30^b^	0.30^b^	0.35^a^	0.01	0.010

Effects of wheat germ diet on MDA and T-AOC levels in the duodenum, jejunum, and ileum. Data are presented as the mean ± SEM (*n* = 4 per group). Different letters denote significant differences between groups (*P* < 0.05), while the same letters indicate no significant difference (*P* > 0.05).

### 3.6 Improving intestinal immunity

The levels of IL-1β, IL-6, and IL-10 in the serum were evaluated. Compared to the control group, the serum IL-6 concentration was decreased by 54.75% in the LWG group, while the IL-1β concentration was increased by 8.7% in the HWG group (*P* < 0.05). However, there were no significant changes in IL-6 and IL-10 levels in the HWG group (*P* > 0.05). Furthermore, the intestinal immunity was evaluated by examining serum IgA and IgG levels (Table 7), as well as intestinal sIgA content (Table 8). Serum IgA and IgG levels remained unchanged among the three treatment groups (*P* > 0.05). Among the three treatment groups, there were no significant changes in intestinal sIgA (*P* > 0.05), but the sIgA levels in the ileum and jejunum were significantly increased in the HWG group (*P* < 0.05).

**TABLE 7:** The levels of IgA, and IgG in serum.

Parameters	Con	LWG	HWG	SEM	*P*-value
IL-1β, pg/mL	76.71^b^	77.44^b^	83.38^a^	1.04	0.010
IL-6, pg/mL	29.08^a^	13.16^b^	28.06^a^	1.93	< 0.001
IL-10, pg/mL	14.63	14.81	14.84	0.30	0.983
IgA, μg/mL	112.94	105.72	108.59	2.29	0.448
IgG, μg/mL	34.76	34.33	35.17	0.81	0.930

Effect of wheat germ diets on serum inflammatory factor, IgA and IgG levels. Data are presented as the mean ± SEM (*n* = 4 per group). Different letters denote significant differences between groups (*P* < 0.05), while the same letters indicate no significant difference (*P* > 0.05).

**TABLE 8:** The levels of sIgA in the duodenum, jejunum, and ileum.

Parameters	Con	LWG	HWG	SEM	*P*-value
Duodenum	5.38	6.07	5.92	0.25	0.670
Jejunum	5.32^b^	5.64^ab^	6.31^a^	0.18	0.060
Ileum	2.27^b^	2.82^b^	3.71^a^	0.12	0.010

Effects of wheat germ diets on sIgA levels in the duodenum, jejunum, and ileum. Data are presented as the mean ± SEM (*n* = 4 per group). Different letters denote significant differences between groups (*P* < 0.05), while the same letters indicate no significant difference (*P* > 0.05).

### 3.7 Promoting intestinal barrier function

To further assess intestinal barrier function, we measured serum LPS levels and DAO activity (Table 9). Compared to the control group, the levels of LPS and DAO activity in the serum were decreased by 11.04 and 26.51% in the LWG group (*P* > 0.05), and the levels of LPS and DAO activity in the serum were decreased by 9.88% and 42.03% in the HWG group (*P* < 0.05).

**TABLE 9:** The levels of LPS and the Activity of DAO in serum.

Parameters	Con	LWG	HWG	SEM	*P*-value
LPS, EU/mL	444.85^a^	395.73^b^	400.91^b^	7.88	0.010
DAO, ng/mL	7.47^a^	5.49^b^	4.33^c^	0.18	< 0.010

Effect of wheat germ diets on serum LPS and DAO. Data are presented as the mean ± SEM (n = 4 per group). Different letters denote significant differences between groups (*P* < 0.05), while the same letters indicate no significant difference (*P* > 0.05).

### 3.8 Improving short-chain fatty acids in cecal metabolism

Table 10 illustrates the changes in short-chain fatty acids (SCFAs) content in cecal contents. Compared to the control group, the levels of acetic acid, butyric acid, and valeric acid were increased by 27.22, 38.89 and 66.67 in the LWG group (*P* < 0.05), whereas the differences in isovaleric and propionic acid contents remained unchanged (*P* > 0.05). Conversely, the acetic acid and propionic acid levels was 20.56 and 17.95% lower in the HWG group than those in the control group (*P* < 0.05), while the levels of butyric acid, isovaleric acid, and valeric acid were unaffected (*P* > 0.05).

**TABLE 10:** Content of short chain fatty acids in cecal contents.

Parameters	Con	LWG	HWG	SEM	*P*-value
Acetic acid, mmol/L	1.80^b^	2.29^a^	1.43^c^	0.11	< 0.001
Propionic acid, mmol/L	0.78^a^	0.92^a^	0.64^b^	0.05	< 0.001
Butyric acid, mmol/L	0.54^b^	0.75^a^	0.47^b^	0.03	0.010
Isovaleric acid, mmol/L	0.07^ab^	0.08^a^	0.63^b^	0.04	0.145
Valeric acid, mmol/L	0.03^b^	0.05^a^	0.030^b^	0.002	0.001

Effect of wheat germ diets on the content of short-chain fatty acids in cecal contents. Data are presented as the mean ± SEM (*n* = 6 per group). Different letters denote significant differences between groups (*P* < 0.05), while the same letters indicate no significant difference (*P* > 0.05).

### 3.9 Improving intestinal flora structure

**FIGURE 2:**
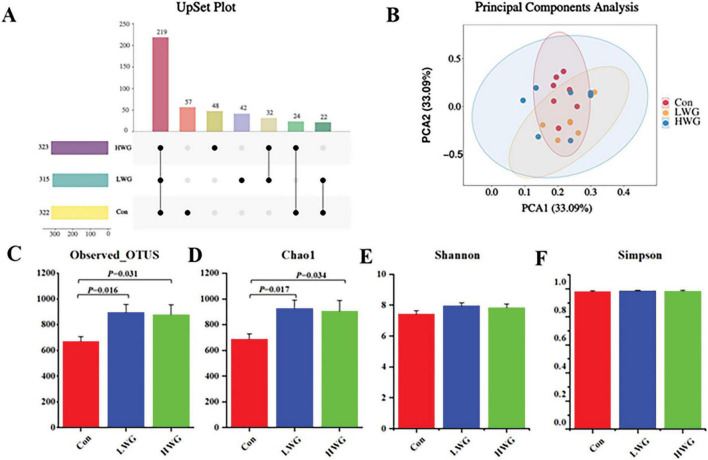
Effect of wheat germ diets on the structure of intestinal flora (*n* = 6). **(A)** OTUs common to or specific to each group. **(B)** PCA analysis of cecal contents flora. Alpha diversity of cecal microflora. Observed Otus **(C)**, Chao1 **(D)**, Shannon **(E)**, Simpon **(F)**. Data are presented as the mean ± SEM (*n* = 6 per group). Different letters denote significant differences between groups (*P* < 0.05), while the same letters indicate no significant difference (*P* > 0.05).

The abundance and diversity of cecal microorganisms were assessed based on gut microbiota sequencing data. A total of 960 OTUs were detected among the 3 treatment groups. Specifically, the Con, LWG, and HWG group had 57, 42, and 48 unique OTUs, respectively (Figure 2A). Principal component analysis (PCA) results showed no significant differences in β diversity between the three groups (Figure 2B). However, the wheat germ groups exhibited significantly higher levels of Observed-OTUs and Chao1 compared to the control group (*P* < 0.05; Figures 2C, D). No significant changes were observed in Shannon and Simpson indices among the three treatment groups (*P* > 0.05; Figures 2E, F).

**FIGURE 3:**
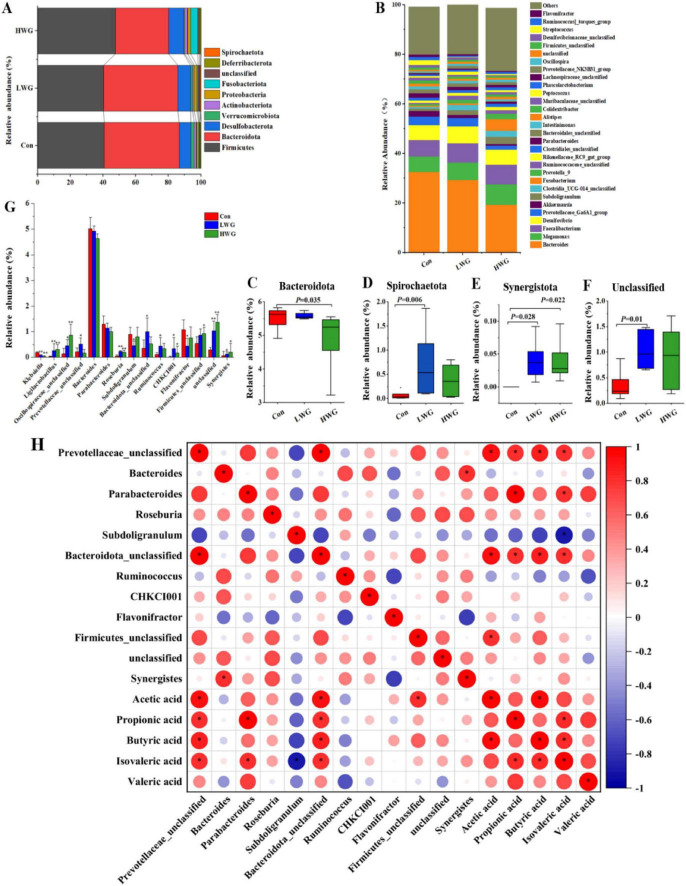
Effect of wheat germ diets on cecum flora. **(A)** Differences in cecum flora at the phylum level. **(B)** Differences in cecum flora at the genus level. **(C–F)** Differences in cecum flora at the phylum level, *Bacteroidota* **(C)**, *Spirochaetota* **(D)**, *Synergistota* **(E)**, *Unclassified* **(F)**. **(G)** Differences in cecum flora at the genus level. Data are presented as the mean ± SEM (*n* = 6 per group). **(H)** A correlogram showing association between cecal SCFAs concentrations and the abundant cecal microbial genera. The Spearman’s correlation coefficient is denoted by the color of the heatmap, with red indicating a positive correlation and bule indicating a negative correlation. The color depth indicates the strength of correlation; the asterisk represents a statistically significant correlation (*P* < 0.05).

The cecum contents were primarily dominated by *Firmicutes*, *Bacteroidota*, *Desulfobacterota, Verrucomicrobiota*, and *Actinobacteriota* at the phylum level (Figure 3A). At the genus level, the dominant gut flora included *Bacteroides*, *Megamonas*, *Faecalibacterium*, *Desulfovibrio*, *Prevotellaceae_Ga6A1_group*, and *Subdoligranulum* (Figure 3B). At the phylum level, the LWG group exhibited a significant increase in the relative abundance of *Spirochaetota, Synergistota*, and various unclassified microbes compared to the control group. The HWG group showed an increase in the relative abundance of *Synergistota*, while the relative abundance of *Bacteroidota* was reduced (*P* < 0.05; Figures 3C–F). At the genus level, the LWG group displayed a significant increase in the relative abundance of *Ligilactobacillus*, *Oscillospiraceae_unclassified*, *Prevotellaceae_unclassified*, *Roseburia*, *Ruminococcus*, *Bacteroidota_unclassified*, *CHKCI001*, and *Synergistes* colonies (*P* < 0.05). However, relative abundance of *Klebsiella*, *Subdoligranulum*, and *Flavonifractor* was markedly decreased (*P* < 0.05). The HWG group showed an increase in the relative abundance of *Ligilactobacillus*, *Oscillospiraceae_unclassified*, *Roseburia*, *CHKCI001*, *Firmicutes_unclassified*, *unclassified*, and *Synergistes colonies* (*P* < 0.05). Additionally, wheat germ significantly increased the relative abundance of unclassified microbes (*P* < 0.05; Figure 3G).

To explore whether the microbiota was associated with SCFAs, a correlation analysis was carried out. Figure 3H that was generated based on the Spearman correlation coefficients, shows the relationships between the genera and the cecal SCFAs changes with wheat germ supplementation. Among them, the relative abundances of *Prevotellaceae_unclassified* and *Bacteroidota_unclassified* were positively correlated with acetic acid, propionic acid, butyric acid, and isovaleric acid (*P* < 0.05); *Parabacteroides* was positively associated with acetic acid, propionic acid, and isovaleric acid (*P* < 0.05); *Firmicutes_unclassified* was positively associated with acetic acid (*P* < 0.05).

### 3.10 Impacting the transcription level of jejunum immune genes

**FIGURE 4:**
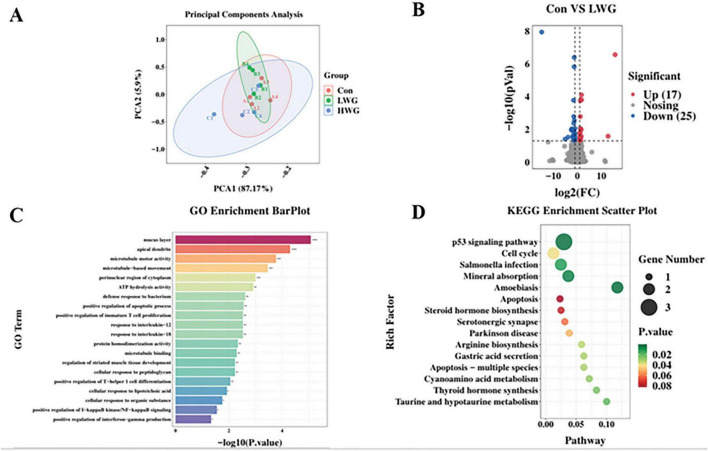
Sequencing of the jejunal transcriptome (*n* = 4 per group). **(A)** RNA-seq correlation test in the three groups. **(B)** Volcano map of differentially expressed genes (LWG vs. Con). Red points represent up-regulated genes with adjusted padj < 0.05 (-log10 (padj) ≥ 1.5). Blue points represent down-regulated genes with padj < 0.05 (-log10 (padj) ≥ 1.5). Black points represent genes with no significant differences. **(C)** Gene ontology (GO) classifications of differentially expressed genes (DEGs) between CT and FOS groups. **(D)** Scatterplot of enriched KEGG pathways for DEGs between LWG and Con groups. Rich factor is the ratio of the DEG number to the total gene number in a certain pathway. The size and color of the dots represent the gene number and range of the FDR, respectively.

Transcriptomic analysis was performed on 12 samples from three groups of Sichuan white goose jejunum. A total of 77.59 Gb of raw sequencing data was obtained, with 73.83 Gb of high-quality reads after filtering. The mapping rate to the Sichuan White Goose reference genome ranged from 81.55 to 85.27%. PCA analysis confirmed good biological replication within the three Sichuan white goose groups (Figure 4A).

Furthermore, differential expression analysis was conducted, and genes were considered differentially expressed if they had a statistical significance of *P* < 0.05 and | log2 (fold change)| > 1.5. Compared to the control group, the LWG group exhibited significant up-regulation of 17 genes, including *CENPF*, *CPED1*, *LXDC2*, *PLXDC2*, *HTRA1*, *CDKL1*, *MGP*, *MMRN1*, *THBS1*, *CEP85*, and *KIF20B.* Additionally, 25 genes, such as *SLC37A1*, *SLC6A4*, *TMIGD1*, *AFP*, *NEURL1*, *IL20RA*, *GGT1*, *OTOP2*, *RGSL1*, *CLCN1*, *PMAIP1*, *SLC39A4*, *MAP3K8*, *TSPAN1*, *and NOS2* were significantly down-regulated in the LWG group (Figure 4B). GO enrichment pathway analysis revealed that the LWG group was mainly enriched in processes such as the mucus layer, apical dendrite, microtubule motor activity, microtubule-based movement, ATP hydrolysis activity, regulation of immune system process, protein homodimerization activity, response to interleukin-18, response to interleukin-12, positive regulation of immature T cell proliferation, defense response to bacterium, and positive regulation of apoptotic process compared to the control group (Figure 4C). Notably enriched KEGG pathways included the p53 signaling pathway, cell cycle, Salmonella infection, mineral absorption, and amoebiasis (Figure 4D).

## 4 Discussion

### 4.1 Effects of wheat germ on intestinal morphology and digestive capacity

The integrity of the intestinal morphology plays a critical role as a physical barrier against external pathogens and serves as the physiological foundation for digestion and absorption (Mitchell and Carlisle, 1992). The presence of well-developed intestinal villi is an important indicator of intestinal integrity and efficient nutrient utilization. An increase in intestinal VH indicates an expansion of the surface area, while an elevated ratio of VH to CD indicates enhanced nutrient absorption capacity (Tufarelli et al., 2010; Li et al., 2022). In this study, the inclusion of wheat germ in the diet resulted in significant improvements in duodenum and ileum VH and VH/CD, significantly reduced the duodenum and ileum CD ratio in both the HWG and LWG groups. These findings suggest that wheat germ promotes intestinal development and improves the functionality of intestinal digestion and nutrient absorption. By enhancing intestinal morphology, wheat germ can potentially optimize the utilization of nutrients, leading to improved production performance and overall health status in poultry.

Intestinal digestive enzyme activity serves as a valuable indicator of digestive and metabolic capacity. Trypsin, lipase, and amylase are key digestive enzymes associated with the breakdown and utilization of major nutrients (Yang et al., 2017; Bedford and Apajalahti, 2022). Wheat germ is known to be rich in various enzymes, including α-amylase, lipase, lipoxygenase, and protease (Kublicki et al., 2022). Our findings demonstrated that amylase and lipase activities were significantly increased in the duodenum and ileum, and increased trypsin activity was observed in the jejunum of the LWG and HWG groups. Previous studies have suggested that enhanced intestinal digestive enzyme activity promotes the efficient digestion and absorption of nutrients in food (Lu et al., 2023). Therefore, our results indicate that wheat germ can increase the digestive enzyme activity in the intestinal tissue of Sichuan white geese, leading to improved nutrient utilization.

### 4.2 Effects of wheat germ on intestinal polyamines levels

Polyamines are essential for maintaining intestinal homeostasis (Yu et al., 2023). Previous studies have shown that inhibition of intestinal polyamine activity can disrupt the integrity of the intestinal epithelium and impair barrier function. During inflammatory enteritis or intestinal mucosal injury, the level of polyamines in mucosal tissue undergoes significant changes, and polyamines are known to promote the proliferation and differentiation of intestinal epithelial cells in animals (Jainu et al., 2010; Ray and Johnson, 2014). Supplementation of exogenous spermidine has been shown to alleviate metabolic endotoxemia, enhance intestinal barrier function, reduce body weight, and ameliorate high-fat diet-induced obesity in mice (Ma et al., 2020). Polyamines have also been found to promote the intestinal development of piglets and regulate jejunum VH and CD in mice (Liu et al., 2019; Jiang et al., 2023). In this study, the levels of putrescine and spermine in the duodenum in the LWG group increased, while spermidine levels showed no significant difference. These findings differ from those of the above studies, this difference could be attributed to variations in diet composition and the differential absorption of spermidine in the intestine. Our results demonstrate that wheat germ diets significantly affected intestinal polyamine metabolism, leading to increased levels of polyamines in the intestine.

### 4.3 Effects of wheat germ on intestinal antioxidant capacity

Wheat germ contains various bioactive components, such as polyamines and phenols, that exhibit strong reactive oxygen species scavenging and antioxidant properties. Studies have shown that wheat germ extract can effectively inhibit reactive oxygen species increase in LPS-induced porcine intestinal epithelial cells and alleviate oxidative stress (Karancsi et al., 2020). Additionally, wheat germ has been found to significantly decrease MDA levels in the intestinal tissue of rats fed a high-fat diet, while increasing SOD activity and total antioxidant capacity (Liu et al., 2022). In line with these findings, this study revealed that intestinal MDA levels were significantly reduced and total antioxidant capacity was enhanced in the duodenum and ileum in the LWG and HWG groups. These results suggest that the inclusion of wheat germ in the diet improves intestinal health by boosting the overall antioxidant capacity of the intestine, thus maintaining a balanced oxidative stress and antioxidant system.

### 4.4 Effects of wheat germ on intestinal physical barrier

Maintaining the integrity and proper morphology of the intestine is crucial for its normal physiological functioning, as disruption of the intestinal barrier can lead to a variety of gastrointestinal diseases. The tight junctions between intestinal epithelium cells prevent the penetration of toxic and harmful substances into surrounding tissues. The impaired intestinal barrier can lead to chronic inflammation and activation of immune cells (Zhu et al., 2024). Recent studies have shown that the intestinal barrier can be used as a potential immunoregulatory target to regulate and prevent different inflammatory diseases through dietary interventions, although the specific regulatory mechanisms remain unclear (Dang et al., 2023). Endotoxin, a key component of the cell wall of Gram-negative bacteria in the intestine, is released when intestinal permeability is compromised, which in turn triggers an inflammatory response in the host (Alexander and Rietschel, 2001). Previous studies have shown that a high-fat diet induces intestinal permeability and increases LPS levels in mice (Wang et al., 2024). However, exogenous spermidine has been shown to significantly enhance the number of mucus-secreting goblet cells and mucin secretion in colon tissue, leading to reduced serum LPS levels (Zhou et al., 2024). Transcriptome sequencing further revealed the suppression of inflammation-related genes and the up-regulation of autophagy and barrier maintenance genes (Ma et al., 2020). These findings indicate that spermidine can strengthen the intestinal barrier, preventing the entry of LPS into the bloodstream and mitigating inflammatory responses in the host. In this study, we found that serum LPS levels and amine oxidase activity were decreased in the LWG and HWG groups. This indicates that wheat germ exhibits similar effects to spermidine by reducing intestinal permeability, preserving intestinal barrier function, and maintaining overall host health.

### 4.5 Effects of wheat germ on immune barrier

SIgA, IgA, and IgG are the major immunoglobulins present in the intestine, and a decrease in their levels can impair intestinal anti-infection ability (Sylvestre et al., 2023). Previous studies have shown that wheat germ can reverse cyclophosphamide-induced immune disorders in mice and restore their immune function (Yu G. et al., 2021). Wheat germ glycoprotein has been found to promote the secretion of sIgA without significantly affecting serum IgA in neonatal mice (Yun et al., 2021). Cytokines such as IL-1β, IL-6, and IL-10 play crucial roles in modulating the innate immune system (Wang et al., 2023b). In a high-fat diet-induced inflammation model, wheat germ was observed to significantly reduce serum levels of IL-1β and IL-6 in mice, while having no effect on IL-10 (Ojo et al., 2019). Moreover, wheat germ significantly reduced pro-inflammatory cytokine-17 levels in serum and ileum of IL-10 knockout mice induced by a Pro-Atherogenic diet, increased IL-22 gene expression and its receptor IL-22α in the ileum, and up-regulated the expression of tight junction proteins, indicating that wheat germ can inhibit pro-inflammatory cytokine-17 through IL-22 signaling pathway (Alake et al., 2023). The above results are consistent with our findings. Our results showed that serum IL-6 was reduced in the LWG group but there was no effect on serum IL-1β, IL-10, IgA, and IgG, or intestinal sIgA levels. In the HWG group, sIgA levels in the jejunum and ileum were increased, while serum IL-6 and IL-10 levels remained unchanged. This may be because a variety of immune cells are involved in the production of IL-10, and wheat germ have limited effects on these immune cells that regulate IL-10 production, so they do not affect the levels of the inflammatory factor IL-10 in serum. This also proves that the decrease of pro-inflammatory factor IL-6 in serum is not due to the increase of anti-inflammatory factor IL-10. In this experiment, the regulation trend of immune function in geese was not consistent between the LWG group and HWG group. We speculate that this inconsistency may be attributed to the large variation in the content of individual components present in different doses of the wheat germ diet. Therefore, further investigations are necessary to elucidate the precise mechanism of action.

Studies have shown that wheat germ effectively regulates the expression of immune factors in the intestine, such as cytokines IL-2 and TNF-α (Yun et al., 2020). This regulation involves the promotion of the CD40L and NF-κB-p65 expression, ultimately leading to improved intestinal immunity from the early stages of development to adulthood via the CD40L-CD40-IKKα/β-NF-κB p65 signaling pathway (Dang et al., 2023). Our transcriptomic sequencing analysis further supports the notion that wheat germ regulates the transcriptional levels of immune genes in the intestine. For instance, THBS1 acts as an intracellular chaperone, facilitating ER responses and enhancing the activity of the secretory pathway and extracellular matrix production. Moreover, THBS1 aids in the retention of adherents on the cell membrane. In the context of heart health, overexpression of THBS1 has been found to have a protective effect (Schips et al., 2019). Another gene of interest-IL20RA, has been involved in the development and progression of colorectal cancer (CRC). Its involvement may stem from its impact on oxygen binding, oxygen transport, and hormone activity. In fact, the knockdown of IL-20RA has been shown to inhibit the growth and metastasis of CRC (Liu et al., 2021; Yu D. Y. et al., 2021).

We identified several DEGs associated with cell cycle and immunity, including *CENPF*, *CDKL1*, *CLCN1*, *CDKL1*, *PLXDC2*, *MMRN1*, *THBS1*, *FABP6*, *TMIGD1*, *IL20RA*, *CLCN1*, *PMAIP1*, and *MAP3K8*. Functional analysis revealed the enrichment of pathways such as the p53 signaling pathway, cell cycle, and immunomodulation-related pathways. The p53 signaling pathway is involved in DNA repair to prevent mutations resulting from DNA damage and plays a crucial role in apoptosis induction, thereby maintaining normal tissue and organ function (Hernandez Borrero and El-Deiry, 2021; Wang et al., 2023a). Wheat germ upregulated the expression of genes *THBS1* and *CDKL1* while down-regulating the expression of genes *IL20RA* and *MMRN1*, suggesting that wheat germ may influence the regulation of cell cycle and immune transcription levels in animals. Nevertheless, our findings indicate that wheat germ may have an important role in the regulation of body immunity and prevention of disease.

### 4.6 Effect of colony structure and metabolites

The intestinal flora has many physiological functions such as enhancing host immunity, facilitating nutrient absorption, and resisting pathogenic microorganisms (Gebrayel et al., 2022). Studies have confirmed that wheat germ supplementation can reshape the structure of intestinal flora. For instance, in mice, wheat germ has been found to reduce the abundance of the *Bacteroidetes* phylum while increasing the levels of SCFA-producing genera such as *Roseburia* (Yu G. et al., 2021). Wheat germ supplementation selectively increased the population of lactic acid bacteria in the intestines of mice on a high-fat diet and enhances the production of antimicrobial peptides in the ileum (Ojo et al., 2019). *Ligilactobacillus* is a lactic acid bacterium with antibacterial and immunomodulatory effects (Widyastuti et al., 2021; Guerrero Sanchez et al., 2022). Oral administration of *Lactobacillus* to mice with colitis has been found to decrease inflammatory factors in their serum, contribute to the restoration of intestinal barrier function, and improve the structure of the bacterial flora (Yao et al., 2021). Additionally, the metabolite butyrate and its associated proteins have been shown to regulate barrier homeostasis and inhibit intestinal inflammation, while whole grain consumption has been shown to improve intestinal metabolic disorders by regulating the abundance of *Roseburia* intestinalis (Martinez et al., 2013; Nie et al., 2021). *Oscillospiraceae_unclassified* has been found to decrease inflammatory factors in serum and enhance intestinal tight junction proteins. On the other hand, Klebsiella is a conditionally pathogenic bacterium known to cause various infections (Yang et al., 2022). In this study, the consumption of wheat germ (LWG group) increased the relative abundance of *Bacteroidota_unclassified*, *Ligilactobacillus*, *unclassified*, and *Roseburia*, while decreasing the relative abundance of *Klebsiella*, *Subdoligranulum*, and *Flavonifractor*. Furthermore, the wheat germ diet led to an increase in the relative abundance of *Oscillospiraceae_unclassified, Firmicutes_unclassified, Roseburia*, *Ligilactobacillus*, and *unclassified* bacteria in the HWG group. These findings indicate that wheat germ promotes the growth of beneficial intestinal bacteria while reducing the presence of harmful bacteria, thus maintaining the structural balance of the gut microbiota and improving intestinal health.

SCFAs play an important role in regulating host energy metabolism, intestinal morphology, and immune function, they have the ability to modulate various aspects of mucosal homeostasis, including the intestinal barrier, T cell populations, and the expression of many inflammatory cytokines (Anachad et al., 2023). Dietary interventions that alter SCFA levels have shown promise in preventing and treating diseases (Portincasa et al., 2022; Tan et al., 2023). For instance, Li et al. (2023) treated mice with Pulsatilla chinensis saponin for colitis and found improved colitis symptoms, attributed to increased intestinal SCFA levels and decreased levels of IL-1β, IL-6, and TNF-α. In this study, we found that the levels of acetic acid, butyric acid, and valeric acid were significantly higher in the LWG group compared to the control group. This suggests that wheat germ diets have the potential to maintain intestinal homeostasis and promote intestinal health by increasing the content of SCFA in the intestine.

Unfortunately, this study has some limitations. Firstly, we are currently unable to determine which bioactive substances in wheat germ play a key role in these effects, our study utilized whole WG, which has other bioactive ingredients (e.g., polyamines, carotenoids, tocopherols, flavonoids, policosanols, and phytosterols) that possess immunomodulatory, antioxidant and regulate the intestinal flora structure functions. Secondly, the constraints of experimental resources resulted in a limited number of test animals. Future research needs to further clarify the key components in wheat germ that play a role and to increase the number of experimental animals and the number of sample analyses to more definitively determine the beneficial effects of wheat germ diets on intestinal health.

## 5 Conclusion

**FIGURE 5:**
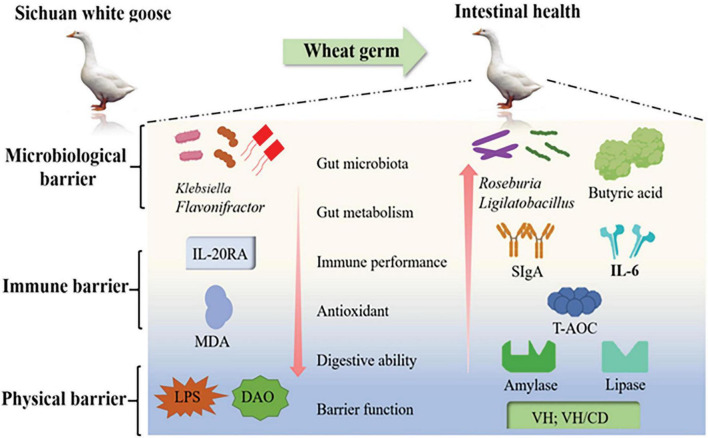
Wheat germ diets have a positive impact on various aspects of intestinal health. These include the maintenance of barrier function, improvement in digestive capacity, enhancement of antioxidant capacity, modulation of immune responses, and regulation of inflammatory processes.

Our findings highlight the beneficial effects of a wheat germ diet on multiple aspects of intestinal health. Specifically, the inclusion of wheat germ in the diet (LWG group) resulted in an increased intestinal VH/CD ratio, as well as enhanced amylase and lipase activities in the duodenum and ileum (*P* < 0.05). Moreover, wheat germ supplementation led to an increase in the levels of putrescine and spermidine in the jejunal region, indicating improved intestinal polyamine levels. Additionally, the LWG group exhibited enhanced total antioxidant capacity and reduced levels of MDA, LPS, and DAO activity in the serum (*P* < 0.05), indicating reduced oxidative stress and improved intestinal barrier function. Furthermore, the inclusion of wheat germ in the diet resulted in favorable changes in the gut microbiota composition. The LWG group showed increased relative abundances of beneficial bacteria, as well as higher levels of acetic acid, butyric acid, and valeric acid. Conversely, the relative abundances of harmful bacteria were decreased. These alterations in the gut flora were associated with changes in immune and cell cycle-related gene expression (*P* < 0.05). Overall, wheat germ diets, particularly the LWG diet have demonstrated a positive impact on various aspects of intestinal health. These include the maintenance of barrier function, improvement in digestive capacity, enhancement of antioxidant capacity, modulation of immune responses, and regulation of inflammatory processes. Additionally, wheat germ diets contribute to favorable changes in the structure and metabolism of the gut microbiota (Figure 5).

## Data Availability

The data presented in the study are deposited in the NCBI Gene Expression Omnibus (GEO), accession number GSE275529.

## Footnotes

1
https://david.ncifcrf.gov/
